# Clinical value of cerebrospinal fluid neurofilament light chain in semantic dementia

**DOI:** 10.1136/jnnp-2018-319784

**Published:** 2019-05-23

**Authors:** Lieke H H Meeter, Rebecca M E Steketee, Dina Salkovic, Maartje E Vos, Murray Grossman, Corey T McMillan, David J Irwin, Adam L Boxer, Julio C Rojas, Nicholas T Olney, Anna Karydas, Bruce L Miller, Yolande A L Pijnenburg, Frederik Barkhof, Raquel Sánchez-Valle, Albert Lladó, Sergi Borrego-Ecija, Janine Diehl-Schmid, Timo Grimmer, Oliver Goldhardt, Alexander F Santillo, Oskar Hansson, Susanne Vestberg, Barbara Borroni, Alessandro Padovani, Daniela Galimberti, Elio Scarpini, Jonathan D Rohrer, Ione O C Woollacott, Matthis Synofzik, Carlo Wilke, Alexandre de Mendonca, Rik Vandenberghe, Luisa Benussi, Roberta Ghidoni, Giuliano Binetti, Wiro J Niessen, Janne M Papma, Harro Seelaar, Lize C Jiskoot, Frank Jan de Jong, Laura Donker Kaat, Marta Del Campo, Charlotte E Teunissen, Esther E Bron, Esther Van den Berg, John C Van Swieten

**Affiliations:** 1 Alzheimer Center and Department of Neurology, Erasmus MC, Rotterdam, The Netherlands; 2 Department of Radiology and Nuclear Medicine, Erasmus MC, Rotterdam, Zuid-Holland, The Netherlands; 3 Penn FTD Center, Department of Neurology, University of Pennsylvania Perelman School of Medicine, Philadelphia, Pennsylvania, USA; 4 Neurology, Memory and Aging Center University of California San Francisco, San Francisco, California, USA; 5 Neurology, University of California San Francisco Memory and Aging Center, San Francisco, California, USA; 6 Alzheimer Center and Department of Neurology, Amsterdam Neuroscience, Vrije Universiteit Amsterdam, Amsterdam UMC, Amsterdam, The Netherlands; 7 Department of Radiology and Nuclear Medicine, Vrije Universiteit Amsterdam, Amsterdam UMC, Amsterdam, The Netherlands; 8 Neurology and Healthcare Engineering, University College London Medical School, London, UK; 9 Department of Neurology, Hospital Clinic de Barcelona, Barcelona, Catalunya, Spain; 10 Institut d’Investigacions Biomèdiques August Pi i Sunyer, Barcelona, Spain; 11 Department of Psychiatry and Psychotherapy, Klinikum rechts der Isar, Technical University of Munich, School of Medicine, Munich, Germany; 12 Clinical Memory Research Unit, Department of Clinical Sciences, Lund University, Lund, Sweden; 13 Psychology, Lund University, Lund, Sweden; 14 Centre for Ageing Brain and Neurodegenerative Disorders, Neurology Unit, Department of Clinical and Experimental Sciences, University of Brescia, Brescia, Italy; 15 Neurodegenerative Diseases Unit, Fondazione IRCCS Ca' Granda, Ospedale Policlinico, Milan, Italy; 16 Biomedical, Surgical and Dental Sciences, University of Milan, Centro Dino Ferrari, Milan, Italy; 17 Pathophysiology and Transplantation, University of Milan, Centro Dino Ferrari, Milan, Italy; 18 Dementia Research Centre, Department of Neurodegenerative Diseases, UCL Institute of Neurology, London, UK; 19 Department of Neurodegenerative Diseases, Hertie Institute for Clinical Brain Research, Tübingen, Germany; 20 German Center for Neurodegenerative Diseases (DZNE), Tübingen, Germany; 21 Institute of Molecular Medicine and Faculty of Medicine, University of Lisbon, Lisbon, Portugal; 22 Department of Neurology, University Hospital Leuven, Leuven, Belgium; 23 Laboratory for Cognitive Neurology, Department of Neurosciences, KU Leuven, Leuven, Vlaanderen, Belgium; 24 Molecular Markers Laboratory, IRCCS Istituto Centro San Giovanni di Dio Fatebenefratelli, Brescia, Italy; 25 MAC Memory Clinic, IRCCS Istituto Centro San Giovanni di Dio Fatebenefratelli, Brescia, Italy; 26 Biomedical Imaging Group Rotterdam, Departments of Medical Informatics and Radiology & Nuclear Medicine, Erasmus MC, Rotterdam, Zuid-Holland, The Netherlands; 27 Imaging Physics, Applied Sciences, Delft University of Technology, Delft, The Netherlands; 28 Department of Clinical Genetics, Leids Universitair Medisch Centrum, Leiden, Zuid-Holland, The Netherlands; 29 Neurochemistry Laboratory, Department of Clinical Chemistry, Amsterdam Neuroscience, Vrije Universiteit Amsterdam, Amsterdam UMC, Amsterdam, The Netherlands

## Abstract

**Background:**

Semantic dementia (SD) is a neurodegenerative disorder characterised by progressive language problems falling within the clinicopathological spectrum of frontotemporal lobar degeneration (FTLD). The development of disease-modifying agents may be facilitated by the relative clinical and pathological homogeneity of SD, but we need robust monitoring biomarkers to measure their efficacy. In different FTLD subtypes, neurofilament light chain (NfL) is a promising marker, therefore we investigated the utility of cerebrospinal fluid (CSF) NfL in SD.

**Methods:**

This large retrospective multicentre study compared cross-sectional CSF NfL levels of 162 patients with SD with 65 controls. CSF NfL levels of patients were correlated with clinical parameters (including survival), neuropsychological test scores and regional grey matter atrophy (including longitudinal data in a subset).

**Results:**

CSF NfL levels were significantly higher in patients with SD (median: 2326 pg/mL, IQR: 1628–3593) than in controls (577 (446–766), p<0.001). Higher CSF NfL levels were moderately associated with naming impairment as measured by the Boston Naming Test (*r_s_*=−0.32, p=0.002) and with smaller grey matter volume of the parahippocampal gyri (*r_s_*=−0.31, p=0.004). However, cross-sectional CSF NfL levels were not associated with progression of grey matter atrophy and did not predict survival.

**Conclusion:**

CSF NfL is a promising biomarker in the diagnostic process of SD, although it has limited cross-sectional monitoring or prognostic abilities.

## Introduction

Semantic dementia (SD) is a sporadic neurodegenerative disorder characterised by loss of semantic knowledge, impaired naming and word comprehension, with preserved speech production.^1^ Compared with other disorders in the frontotemporal lobar degeneration (FTLD) spectrum, SD is relatively homogeneous because of the typical clinical presentation, the neuroimaging signature of asymmetrical anteroinferior temporal atrophy and the typical pathology of type C FTLD with TAR DNA binding protein 43 kDa inclusions (FTLD-TDP).^1–3^ This homogeneity provides opportunities for the development of disease-modifying agents, for which reliable biomarkers are essential to measure their efficacy.

A promising biomarker in frontotemporal dementia (FTD) is neurofilament light chain (NfL), a major component of the neuronal cytoskeleton involved in axonal and dendritic growth, signalling and transport.^4^ Previous studies have demonstrated elevated cerebrospinal fluid (CSF) NfL levels across the FTLD spectrum which are associated with disease severity, brain atrophy and survival.^5–11^ Moreover, CSF and serum NfL levels are strongly correlated, enabling repeated measurements in serum to assess disease progression or treatment response.^5 12^ Small series have shown high CSF and serum NfL concentrations exclusively in the group of patients with SD,^7 13–15^ but a larger cohort may be needed to detect associations with clinical variables. Another interesting question in this context is whether high NfL levels are also associated with survival in SD, considering that SD is a relatively slow progressive disease.^16 17^

In a large series of patients with SD from 14 different centres, we investigated our hypothesis that CSF NfL levels are elevated compared with controls and correlate with disease severity, atrophy and clinical progression in SD.

## Methods

### Subjects

In total, 168 patients with SD with one CSF collection from 14 different centres in Europe and the USA (numbers per site in online supplementary table 1) were retrospectively included in this study. Patients with a CSF profile suggestive of Alzheimer’s pathology (a combination of low amyloid-β_1-42_ and high phospho-tau and/or total-tau,^18^ according to local references at time of CSF collection), were excluded from the study (n=6). Patients initially presented with language difficulties, characterised by fluent speech with impaired naming and word comprehension. The clinical diagnosis of SD was established using the international consensus criteria at the time of inclusion (either based on Neary *et al*. in those cases diagnosed before 2011 or Gorno-Tempini *et al*. from 2011 onwards).^3 19^ Some behavioural disturbances were present in a subset of patients (n=63), but language problems were the most prominent initial features in all. In addition to the clinical diagnosis of SD, the other inclusion criteria were: availability of CSF NfL concentrations (n=162) and survival (n=157), neuropsychological (n=147) and/or neuroimaging data available for analysis (n=87).

10.1136/jnnp-2018-319784.supp1 Supplementary data

Asymmetric temporal atrophy on neuroimaging was used as a supportive feature; this was present in 141 patients (left-sided dominant atrophy in 108 patients; right-sided dominant atrophy in 33 patients). The remaining 21 patients with CSF NfL concentrations fulfilled the clinical diagnostic criteria of SD, but had no neuroimaging available (n=18) or had bilateral atrophy (n=3). TDP-pathology was confirmed in all seven deceased patients with brain autopsy.

To compare NfL levels between controls and patients with SD, 65 sex-matched and age-matched healthy controls from our previous studies were included.^5 7^ Controls had normal CSF amyloid-β_1-42_ levels and had either normal neurological examinations, neuropsychological testing scores and Clinical Dementia Rating (CDR) scores of 0 (n=44) or they were cognitively healthy family members without a mutation or spouses from patients with genetic FTD or a different neurodegenerative dementia (n=21).

Disease duration was defined as time between first symptoms noted by a caregiver (onset) and CSF collection. Survival was defined as time between CSF collection and death.

### Standard protocol approvals, registrations and patient consents

The local ethics committees approved the study and all subjects or their legal representatives provided written informed consent.

### Neuropsychological assessment

**Figure 1:**
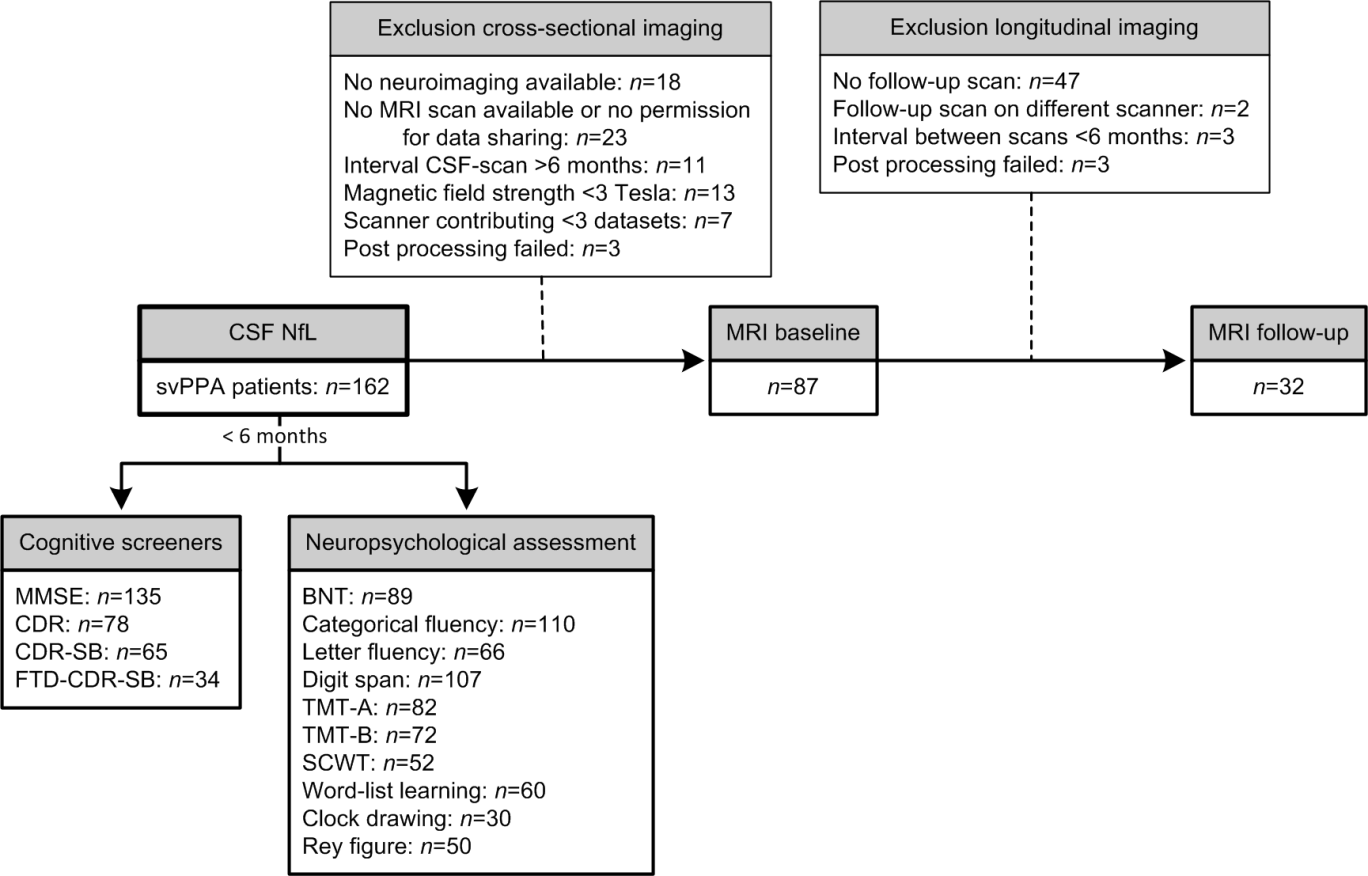
Flowchart of patients with SD included and excluded per analysis. In total, 162 patients with SD were studied after exclusion of six patients with a CSF profile suggestive of Alzheimer’s disease, of which 87 were included in the cross-sectional imaging associations and 32 in the longitudinal imaging associations; reasons for exclusion are displayed in the upper boxes. The number of patients included in the associations with cognitive screeners and neuropsychological tests is displayed in the lower boxes; this is based on the availability of these measures within 6 months of CSF sampling. BNT, Boston Naming Test; CDR, Clinical Dementia Rating scale; CDR-SB, CDR-sum of boxes; CSF, cerebrospinal fluid; FTD, frontotemporal dementia; MMSE, Mini-Mental State Examination; SCWT, Stroop Color-Word Task; SD, semantic dementia; TMT, Trail-making Test.

Most subjects (n=147) underwent global cognitive screening and/or neuropsychological assessment (NPA) at the local study site (for numbers per test, see figure 1); only assessments within 6 months of CSF collection were analysed. Screening instruments included the Mini-Mental State Examination (MMSE), global CDR scale, CDR-sum of boxes (CDR-SB) and the FTD-CDR-SB. When follow-up scores were available, annual progression rate after CSF collection was calculated by the change in MMSE, CDR, CDR-SB or FTD-CDR-SB divided by the number of years between baseline and follow-up (at least 6 months).^20–22^

NPA batteries differed across the sites, and tests were only included when available in at least 30 patients (figure 1). A proportion of the test scores were transformed to uniformly and meaningfully combine different tests or versions, and thus use the maximal amount of neuropsychological data. Short versions of the Boston Naming Test (BNT) were multiplied to match the total possible score of the full 60-item version. Trail-making Test-part A (TMT-A) and part B (TMT-B) were truncated to 300 s for patients that exceeded the time limit of 300 s. For the Stroop Color-Word Test (SCWT), some versions scored the number of correct items within a set time, whereas others obtained the time to complete 50 or 100 items. We transformed all scores into the number of seconds needed to complete a 100-item version; for SCWT versions that scored the number of correct items, we used the following formula: number of seconds allowed * 100/number of correct items. Next, the interference score (score on interference card/score on colour naming) was calculated for all SCWTs and used for analysis. Different word-list learning tasks were transformed into a percentage of correct items (Rey Auditory Verbal Learning Test n=34, California Verbal Learning Test n=24, CERAD word list memory test n=2). In addition, we converted scores on different versions of the Clock Drawing Test (CDT) to percentages. Non-transformed tests included categorical fluency (animal naming), verbal fluency (three letters), digit span (forward+backward) and the Rey complex figure test.

### CSF analyses

CSF was collected and stored according to standardised local procedures. NfL was measured in duplicates by the ELISA (Uman Diagnostics, Umeå, Sweden) according to the manufacturer’s instructions. Measurements were performed in three different laboratories: the Amsterdam University Medical Center (135 patients with SD, 21 controls), Bristol Myers Squib, Wallingford, Connecticut, USA (19 patients with SD and 44 controls) and the Washington University School of Medicine (8 patients with SD). All laboratories used the same ELISA, but the latter two added a dilution step (1:3 diluted, instead of 1:1 as the manual stipulates). Thus, considering the optimal linearity of the assay used,^23^ a correction factor of two was used for all NfL levels determined at these sites, resulting in comparable CSF NfL levels in patients across the different laboratories (p=0.09). Controls from laboratory two had slightly lower NfL concentrations, but with overlapping ranges (laboratory 1 (Amsterdam UMC): median 800 pg/mL, range 548–1093 pg/mL; laboratory 2 (Bristol Myers Squib): median 511 pg/mL, range 99–1047 pg/mL). We covaried analyses for each laboratory. Within all laboratories, the interassay coefficient of variation was within the acceptance criteria (≤20%). The mean intra-assay coefficient of variation was 1.4% (range 0%–11.3%, unavailable in 25 patients).

### Magnetic resonance imaging (MRI)

In 87 patients, structural T1-weighted (T1w) 3T MR-images within 6 months of CSF collection were available for neuroimaging analysis (mean CSF-MRI interval 0±1 months), and in 32 patients a follow-up scan ≥6 months was available (mean interval between scans 13.5±7.0 months). Brain images were acquired locally and scans from a scanner that only contributed one or two scans were excluded, resulting in datasets from nine different scanners; detailed information on numbers and reasons for exclusion are presented in figure 1.

The T1w images were processed according to procedures described by Bron *et al*.^24^ In short, a multiatlas approach was used to calculate grey matter (GM) volume (mL) in 83 regions of interest (ROIs) from each subject’s T1w images. The unified tissue segmentation method^25^ of SPM8 (Statistical Parametric Mapping, London, UK) was used to segment T1w images into GM, white matter and CSF. Then, ROIs were defined for each subject by using a multiatlas approach containing 30 labelled T1w images with 83 ROIs each.^26 27^ A rigid, affine and non-rigid B-spline transformation model was used to register the atlas’ T1w images to the subjects’ T1w images. All images were masked with the brain extraction tool^28^ and non-uniformity corrected, after which a majority-voting algorithm was used to fuse ROI labels. We selected 11 specific cortical ROIs for further analysis from the 83 ROIs previously implicated in SD^1 29 30^ Processed images were visually inspected for each subject and each processing step; six scans had extensive segmentation errors and were therefore excluded (figure 1). For the 11 ROIs, processed images with outlying volumes (<25th percentile – 1.5 * IQR or >75th percentile + 1.5 * IQR) were closely inspected by three raters; if consensus was reached that the outlier was caused by a segmentation error, the specific ROI was excluded from analysis.

Since SD is an asymmetric disorder, GM ROIs of the dominant brain side (most atrophied, based on the smallest temporal lobar GM volume for each patient on the baseline scan) were used for analysis; secondary analyses were undertaken on bilateral GM ROIs. All ROIs were corrected for head size by normalising to intracranial volume (ICV) and reported as percentage of ICV. The degree of asymmetry was assessed by calculating the ratio of temporal lobar GM volume from the dominant side to that of the non-dominant side, yielding a smaller ratio when more asymmetry was present. For follow-up images, progression of atrophy was assessed by change in volume (mL) per year uncorrected for ICV: (volume follow-up – volume baseline)/interval between scans (years).

### Statistical analyses

SPSS Statistics 21.0 for Windows (Armonk, New York, USA) was used to analyse the data. Statistical significance was set at p<0.05 and Bonferroni correction for multiple testing was used when appropriate. Continuous data were compared between two groups by Mann-Whitney U or t-tests where appropriate; categorical variables were compared by χ² tests. CSF NfL levels were normalised by log transformation and compared between patients with SD with controls using analysis of covariance, correcting for sex, age at CSF collection and laboratory of NfL measurement. Diagnostic performance was assessed by receiver operating characteristic analysis, with optimal cut-off levels at the highest Youden index.

Spearman’s correlation coefficient (*r_s_*) was used to correlate non-transformed NfL levels with age at CSF collection, disease duration at CSF collection, cognitive screening scales (MMSE, CDR-SB and FTD-CDR-SB), neuropsychological tests and GM ROIs. Next, multivariate linear regression analysis (*β*) was also used to assess the association between (1) non-transformed NfL levels (independent variable) and neuropsychological tests (dependent variable), correcting for age, sex and laboratory and (2) non-transformed NfL levels and GM ROIs, correcting for age, sex, laboratory and scanner. The Bonferroni method was used for correction for multiple comparisons. NfL as predictor for survival (after CSF collection) in patients was analysed using the Log Rank test and Kaplan-Meier curves comparing NfL tertiles, and a Cox regression with correction for age, sex and laboratory, both on tertiles and NfL as continuous variable.

## Results

### Demographical and clinical data

The group of 162 patients with SD and 65 healthy controls did not differ in age or sex (table 1). The median disease duration at CSF collection in patients with SD was 3.3 years, ranging from 0.3 to 15.2 years. The FTD-CDR-SB scores in patients with SD ranged from 1.0 to 22.0 with a median of 4.0 (n=34). Available neuropsychological data showed poor performances on the BNT (mean=20, n=89) and verbal fluency tasks (semantic fluency: mean=8, n=110; phonological fluency: mean=21, n=66). The median follow-up of living patients was 4.1 years (range 0.6–13.6, n=127; five patients were lost to follow-up) while median survival of deceased patients after CSF collection was 5.3 years (range 1.0–14.1, n=30).

**Table 1:** Subject characteristics

	Patients with SD, n=162	Controls, n=65	P value
Sex, *n* male (%)	75 (46%)	32 (49%)	0.69
Age at CSF collection, years	64 (58–68)	65 (60–70)	0.43
Age at onset, years	60 (54–65)*	n/a	n/a
Age at death, years	69 (65–74)†	n/a	n/a
MMSE score	25 (21–28)‡	n/a	n/a
CDR-SB score	3.5 (2.5–4.8)§	n/a	n/a
FTD-CDR-SB score	4.0 (2.0–6.0)¶	n/a	n/a
CSF NfL, pg/mL	2326 (1628–3593)	577 (446–766)	<0.001
Isolated decreased abeta, *n* (%)	8 (5%)	n/a	n/a
Isolated increased p-tau and/or t-tau, *n* (%)	61 (38%)	n/a	n/a
MRI available at baseline (at follow-up), *n*	87 (32)	n/a	n/a

Continuous variables are presented as medians (IQR).

*Unknown in five patients.

†30 patients were known to be deceased at time of data analysis.

‡Available in 135 patients.

§Available in 65 patients.

¶Available in 34 patients.

CDR-SB, Clinical Dementia Rating scale sum of boxes; CSF, cerebrospinal fluid; FTD-CDR-SB, frontotemporal dementia CDR-SB; MMSE, Mini-Mental State Examination; NfL, neurofilament light chain; SD, semantic dementia; abeta, amyloid-β_1-42_; p-tau and/or t-tau, phospho-tau and/or total-tau.

### NfL in relation to clinical characteristics and neuropsychological test scores

**Figure 2:**
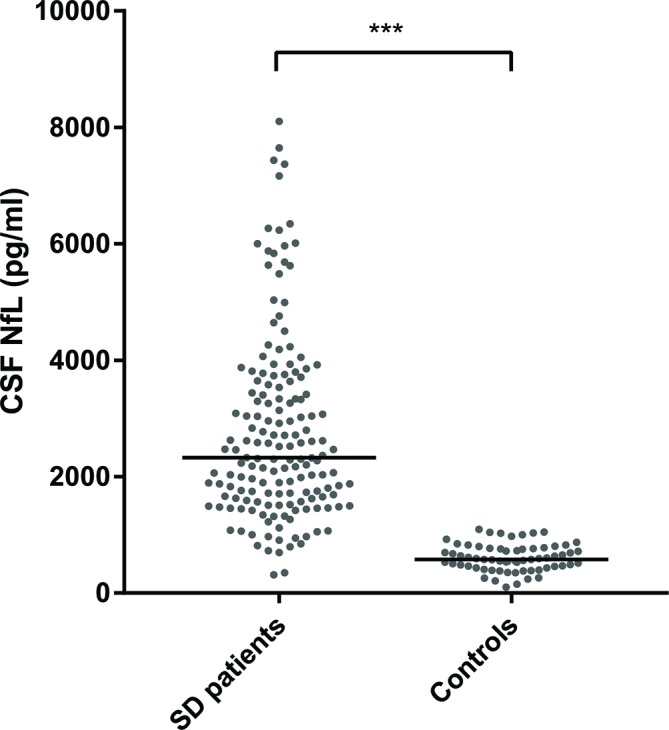
CSF NfL concentrations in patients with SD and controls. The horizontal lines represent the median per group. CSF, cerebrospinal fluid; NfL, neurofilament light chain; SD, semantic dementia; ***p<0.001.

**Figure 3:**
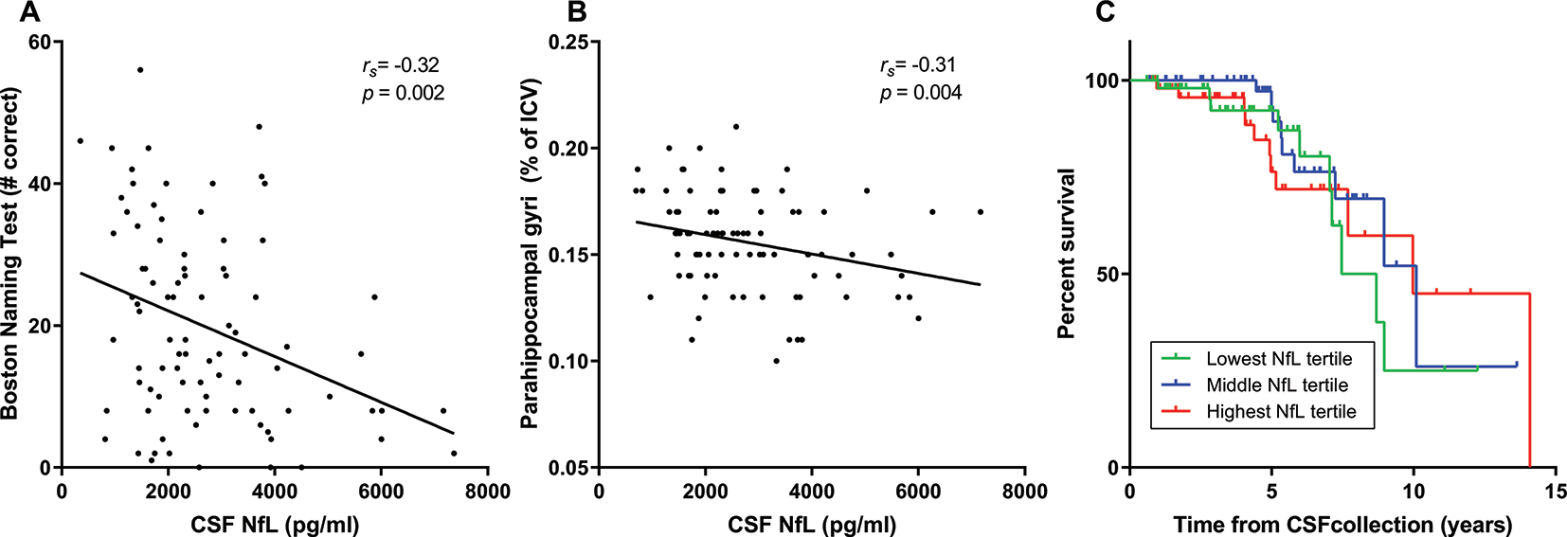
Relationship of CSF NfL with language impairment, parahippocampal atrophy and survival in patients with SD. (A) Association between NfL and the BNT as measure for naming impairment. When the patient (who had a right dominant SD) with a high BNT score was excluded, the analyses remained significant. (B) Association between NfL and grey matter volume of the parahippocampal gyrus at the dominant side, displayed as percentage of ICV. (C) NfL was not associated with survival after CSF collection in SD as exemplified by this Kaplan-Meier curve of NfL levels stratified to lowest (green line), middle (blue line) and highest tertiles (red line). Vertical ticks represent living patients. BNT, Boston Naming test; CSF, cerebrospinal fluid; ICV, intracranial volume; NfL, neurofilament light chain; SD, semantic dementia.

CSF NfL levels were higher in patients with SD than in controls (table 1, figure 2, p<0.001) and had a sensitivity of 93% and a specificity of 98% (cut-off level 1049 pg/mL, area under the curve 0.98 (95% CI 0.96 to 1.00)). Cross-sectional NfL correlated with the BNT (table 2, figure 3A, *r_s_*=−0.32, p=0.002), trended towards an association with annual change of MMSE (table 3, uncorrected p=0.04), but not with age, disease duration at CSF collection, cross-sectional MMSE, CDR-SB or FTD-CDR-SB. TMT-A and TMT-B trended towards association with NfL (table 2), and this was however confounded by age (multivariate regression).

**Table 2:** Associations between neurofilament light chain and neuropsychological test scores

Test		Correlation	Multivariate regression	
	N	r_s_	P value	*β*	P value
BNT	89	−0.32	0.002*	−0.34	0.002*
Categorical fluency	110	−0.17	0.08	−0.14	0.14
Letter fluency	66	−0.03	0.83	−0.14†	0.28
Digit span	107	0.07	0.47	0.04	0.71
TMT-A	84	−0.23	**0.04**	−0.20†	0.07
TMT-B	78	−0.30	**0.007**	−0.19†	0.09
SCWT interference ratio	52	0.12	0.39	0.08‡	0.57
Word list immediate recall	60	−0.17	0.20	−0.22§	0.08
Word list delayed recall	60	−0.23	0.07	−0.24§	0.08
Clock drawing test	30	0.23	0.23	0.21†	0.30
Rey figure copy	50	−0.05	0.71	0.04†	0.76
Rey figure delayed recall	44	−0.17	0.27	−0.24†	0.10

Multivariate regression is corrected for age, gender and laboratory. P<0.05 are in bold.

*Survived Bonferroni correction for multiple testing (p<0.004).

†Not corrected for laboratory as all samples were analysed in the same laboratory for these tests.

‡After additional correction for version seconds to complete versus number correct: β=0.15, p=0.27.

§Similar results with additional correction for test version.

BNT, Boston Naming Test; SCWT, Stroop Color-Word Task; TMT, Trail-making Test.

**Table 3:** Association between neurofilament light chain and clinical characteristics or global cognitive scales in patients with SD

	Cross-sectional			Longitudinal		
	N	*r_s_*	P value	N	*r_s_*	P value
Age at CSF collection	162	−0.002	0.98	n/a	n/a	n/a
Age at onset	157	−0.06	0.46	n/a	n/a	n/a
Disease duration at CSF collection	157	0.03	0.68	n/a	n/a	n/a
MMSE	135	−0.11	0.20	51	−0.29	**0.04**
Global CDR	78	0.15	0.18	33	−0.03	0.86
CDR-SB	65	0.23	0.07	33	−0.01	0.97
FTD-CDR-SB	34	0.05	0.79	10	0.33	0.37

For longitudinal analysis, annualised change of the scores was used. P<0.05 are in bold, none survived Bonferroni correction for multiple testing.

CDR, global score of clinical dementia rating scale; CDR-SB, clinical dementia rating scale sum of boxes; CSF, cerebrospinal fluid; FTD-CDR, frontotemporal dementia clinical dementia rating scale; MMSE, Mini-Mental State Examination.

### Association between NfL and regional GM volumes

CSF NfL levels were negatively associated with GM volume of the parahippocampal gyrus on the dominant atrophic side (table 4, figure 3B, *r_s_*=−0.31, p=0.003), and there was a trend for association with GM volume of the medial and inferior temporal gyri (*r_s_*=−0.28, p=0.06). Cross-sectional NfL levels did not correlate with progression of atrophy in the selected ROIs in the small series of patients with SD with follow-up scans (n=32) (table 4). When the analyses were repeated with bilateral volumes, a negative association with the parahippocampal gyri was found once again (*r_s_*=−0.32, p=0.003). Additionally, the bilateral medial and inferior temporal gyri, temporal pole and hippocampus were negatively associated with NfL; however, after correction for covariates, the association did not withstand Bonferroni correction.

**Table 4:** Associations between neurofilament light chain and grey matter regions of interest of the dominant side*

Region of interest	Cross-sectional (n=87)				Longitudinal (n=32)			
	Correlation		Multivariate regression		Correlation		Multivariate regression	
	r_s_	P value	β	P value	r_s_	P value	β	P value
Temporal pole†	−0.13	0.24	−0.13	0.27	−0.11	0.57	−0.11	0.63
Medial and inferior temporal gyri	−0.28	**0.009**	−0.21	0.06	0.03	0.88	−0.29	0.16
Superior temporal gyrus, central part	−0.17	0.11	−0.16	0.16	−0.01	0.94	−0.07	0.72
Fusiform gyrus	−0.16	0.14	−0.18	0.12	−0.33	0.07	−0.34	0.08
Parahippocampal gyrus	−0.31	**0.004‡**	−0.33	**0.003‡**	0.08	0.67	−0.04	0.85
Hippocampus	−0.20	0.07	−0.20	0.08	0.02	0.93	0.06	0.80
Amygdala	−0.18	0.09	−0.19	0.09	0.12	0.54	0.08	0.69
Insula	−0.19	0.07	−0.17	0.13	−0.09	0.62	0.04	0.82
Orbitofrontal cortex§	−0.05	0.62	−0.05	0.67	0.10	0.59	0.17	0.40
Inferior frontal gyrus	−0.03	0.78	0.04	0.76	0.09	0.61	0.03	0.87
Anterior cingulate gyrus¶	−0.02	0.89	0.07	0.56	0.00	1.00	−0.02	0.91

Association of neurofilament light chain with gray matter regions of the dominant side on cross-sectional scanning and with change in volume between follow-up and baseline scan.

For cross-sectional associations, intracranial volume-corrected volumes were used; for longitudinal associations, the change of volume per year was used. Multivariate regression was corrected for age, gender, laboratory and scanner (the latter only for cross-sectional scans); P<0.05 are in bold.

*Of the patients with a usable baseline MRI scan (n=87), 65 were left-dominant while 22 were right-dominant, which indicated a strong temporal asymmetry (mean ratio temporal atrophy of 0.76 (±0.08).

†Combination of the medial anterior temporal lobe, lateral anterior temporal lobe and the anterior part of the superior temporal gyrus.

‡Survived Bonferroni correction for multiple testing (p<0.0045).

§Combination of the anterior, medial, lateral and posterior orbital gyri.

¶Combination of the supragenual, subgenual and presubgenual part of the anterior cingulate gyrus.

### Survival analyses

Cox regression analysis with cross-sectional CSF NfL as a continuous variable showed a trend for association between NfL levels and survival (p=0.06, HR 1.22 (95% CI 0.99 to 1.51)). However, CSF NfL tertiles did not associate with survival after CSF collection (figure 3C, p=0.66 log rank test, p=0.80 Cox regression with correction for sex, age and laboratory). Excluding patients with an outlying long disease duration (>Q3+1.5*IQR) yielded the same results.

## Discussion

This large international multicentre SD study shows that the clinical value of cross-sectional CSF NfL differs from other FTLD subtypes noted in previous reports. Increased CSF NfL levels in patients with SD were associated with more severe naming impairment and smaller GM volume of the parahippocampal gyrus, both with a medium effect size (correlation coefficient of −0.3). In contrast to other FTLD subtypes, NfL did not associate with progression of GM atrophy or survival.

The elevated CSF NfL levels in patients with SD compared with controls is in line with those found in previous smaller SD series.^7 12–14^ The absence of a correlation between CSF NfL and age is probably caused by the disease effect overriding the age effect. The observed association between higher NfL levels and worse performance on the BNT may suggest that the variance in NfL is partly determined by disease severity, as naming impairments and semantic deficits are hallmarks of SD.^22 31 32^ Future studies will benefit from more extensive and more uniform testing of word comprehension.

While earlier studies found an association of NfL with CDR in different FTD types,^5 7^ we found no association with the CDR in our study. This may be explained by the fact that patients with problems confined to language remained independent in daily life activities.^33^ The FTD-CDR-SB does include a language domain as one of the eight items, but the number of patients with data on this scale was too small to draw strong conclusions. Likewise, the association between NfL and future decline in MMSE did not survive multiple testing correction and seems—combined with its medium effect size—therefore not suitable as a predictor for functional decline.

**Figure 4:**
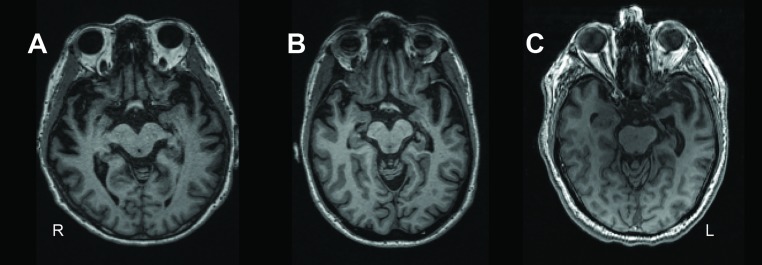
Temporal pole atrophy in patients. Transversal T1-weighted MR images of patients representative for the distribution of dominant anterior temporal pole atrophy within the sample. From left to right: patient with lower quartile (A), median (B) and upper quartile (C) anterior temporal pole grey matter volumes of the dominant side.

The significant association between higher CSF NfL levels and smaller parahippocampal GM volume in our study is in line with studies showing positive correlations with temporal cortical regions in FTD.^5 7^ This is in line with a large body of evidence that NfL levels reflect the extent of neuronal loss^5 7 34^ as supported by neurodegenerative mouse models.^35^ Of note, one previous study using blood found no association of GM volume with serum NfL in patients with SD,^14^ which could be explained by the larger power of our study, or that CSF NfL might be more sensitive than serum NfL.^34^ The parahippocampal gyrus is located in the core of the neurodegenerative process, which starts in the temporal pole and fusiform gyrus spreading to the orbitofrontal, inferior frontal, insular and anterior cingulate cortices as well as posteriorly to temporoparietal regions, and into homologous areas of the contralateral hemisphere.^1 29 30^ More regions were (borderline) significantly associated when analysing bilaterally rather than at the dominant side only. This may point towards an important difference between these markers: GM atrophy represents cumulative injury, while CSF NfL measures the current balance between release and clearance of NfL and thus reflects ongoing neuronal loss. Our notable lack of association of NfL with the anterior temporal lobe may then be explained by the striking atrophy at time of presentation (as illustrated by figure 4) precluding further release of neurofilaments from this region. Other studies report that NfL levels are elevated during active periods of multiple sclerosis and traumatic brain injury and normalise afterwards or after treatment.^36 37^ If this hypothesis on NfL release is correct, the change of NfL over time would associate with progression of atrophy, as has previously been shown for primary progressive aphasia or FTD subtypes combined.^5 13 14^ We found no association between baseline NfL and progression of GM atrophy. This may be due to the relatively small subset of patients with longitudinal MRI data and/or to the slow decrease of GM atrophy over time. Larger series of longitudinal NfL levels with corresponding MRI scans are needed to elucidate these discrepancies, preferably including patients early in their disease process with multiple time-points, and by taking a possible non-linear relationship into account.

We showed a trend for a significant association between cross-sectional NfL levels and future survival in SD. This seems to be in contrast with the evidence of an association between high NfL levels and shorter survival in FTD, progressive supranuclear palsy, Alzheimer’s disease (AD) and amyotrophic lateral sclerosis.^5 6 10 11^ However, the median follow-up time of the entire cohort in our study was 4.3 years (IQR: 2.6–6.0), which is relatively short considering average survival which ranges from 9 to 12 years in SD.^16 17^ Since the follow-up time may have been too short to capture the differences in survival, we propose to reinvestigate the survival of the current cohort in approximately 3–5 years.

A major strength of this study was the large international series of CSF samples and clinical data from patients with this rare disease, which comprises 10%–15% of FTD cases.^16^ A second strength was the multimodal approach in correlating NfL to clinical, neuropsychological and imaging data. Moreover, we included only one FTD subtype—after exclusion of patients with CSF profiles suggestive of AD, making the results specific and not influenced by clinical or pathological heterogeneity.

Since SD is one of the most homogeneous subtypes of FTD, it is a promising target for developing novel treatments. Our results are also important for future trials, as they suggest a limited role for cross-sectional CSF NfL as a monitoring or progression marker in SD.

The multicentre approach also caused some limitations, especially considering the measurement of NfL levels at three different centres. Unfortunately, no controls are provided in the ELISA kit, and due to the current lack of a quality control programme, the assays of the different laboratories could not be compared directly. We accounted for differences between laboratories by normalisation and laboratory-correction, but in future multicentre studies, harmonisation of results could be improved by remeasuring a number of control and patient samples across the different laboratories.

Other possible limitations include the different neuropsychological test batteries per centre and this might have introduced variability and loss of sensitivity; however, we transformed different versions of the same test to analyse them together. Furthermore, the variation in scanners and parameters of the T1-weighted imaging was reduced by including 3T data only, excluding data from scanners on which less than three datasets were acquired and correcting for scanner in the analysis. Additionally, we did not study different primary progressive aphasia subtypes and did not include serum—which is likely to replace CSF measurements in the near future. The lack of longitudinal NfL data did not allow us to draw conclusions about NfL and clinical or imaging markers over time; it is conceivable that a plateau phase or even a decrease of NfL may occur over time.

In conclusion, our results show that cross-sectional elevated CSF NfL may be a useful biomarker for the neurodegenerative process in SD, which could lead to the use of CSF NfL as a diagnostic biomarker. However, the use of CSF NfL to monitor disease progression in SD remains debatable, since we only found a moderate association with language deterioration and atrophy and no relation of CSF NfL with survival (in a relatively short follow-up time). This is in contrast with previous reports on NfL in other neurodegenerative diseases^5 6 10 11^ and should thus be taken into account when interpreting studies that combined different FTLD subtypes. Recently, it has become clear that NfL in blood and CSF strongly correlate,^5 12^ facilitating longitudinal monitoring. More longitudinal multicentre studies are needed to assess how serial NfL levels fluctuate over time in relation to longitudinal clinical and imaging changes in SD (and other FTLD subpopulations) and thereby their potential utility.
