# Supplementary material for: Clinical value of cerebrospinal fluid neurofilament light chain in semantic dementia
Source: J Neurol Neurosurg Psychiatry. 2019 May 23;90(9):997–1004. doi: 10.1136/jnnp-2018-319784 (PMC6820157; doi:10.1136/jnnp-2018-319784)
Supplement: Supplementary data [file jnnp-2018-319784supp001.pdf]

Supplementary Table 1. Number of subjects included per site.

Site	Semantic dementia patients	Controls
University of Pennsylvania	31	0
University of California, San Francisco	27	44
Hospital Clínic Barcelona	14	13
Erasmus Medical Center	17	8
VU University Medical Center	22	0
Tehnical University of Munich	13	0
Lund University	11	0
University of Brescia	7	0
University of Milan	6	0
University College London	6	0
University of Tübingen	3	0
University of Lisbon	2	0
University Hospital Leuven	2	0
IRCCS Centro San Giovanni di Dio Fatebenefratelli	1	0
